# Long-term outcome of neoadjuvant tislelizumab plus chemotherapy in locally advanced esophageal squamous cell carcinoma

**DOI:** 10.1007/s00262-025-04240-8

**Published:** 2025-12-18

**Authors:** Hao Yin, Xinyu Yang, Yujie Chen, Hongtao Duan, Jie Wang, Xuqiang Liao, Guangyu Yao, Fei Liang, Hong Fan, Gao Li, Yihua Sun, Xiaolong Yan, Peiyuan Wang, Lijie Tan

**Affiliations:** 1 Department of Thoracic Surgery, Zhongshan Hospital, Fudan University, Shanghai, 200000 People’s Republic of China; 2 Department of Thoracic Oncology Surgery, Fujian Cancer Hospital, Fuzhou, 35001 People’s Republic of China; 3 Department of Thoracic Surgery, Tangdu Hospital, Air Force Military Medical University, Xi’an 710038, People’s Republic of China; 4 Department of Thoracic Surgery, Fudan University Shanghai Cancer Center, Shanghai, 200032 People’s Republic of China; 5 Department of Thoracic Surgery, Hainan General Hospital, Hainan Affiliated Hospital of Hainan Medical University, Haikou, 570311 People’s Republic of China; 6 Department of Biostatistics, Zhongshan Hospital, Fudan University, Shanghai, 200000 People’s Republic of China; 7 Department of Thoracic Surgery, Zhongshan Hospital, Fudan University (Xiamen Branch), Xiamen, 361015 People’s Republic of China

**Keywords:** Neoadjuvant, Tislelizumab plus chemotherapy, Advanced esophageal squamous cell carcinoma

## Abstract

**Background:**

Current understanding of neoadjuvant immunochemotherapy for locally advanced esophageal squamous cell carcinoma (ESCC) lacks high-level evidence. This study provides additional efficacy data for this treatment regimen.

**Methods:**

Clinical trials investigating neoadjuvant tislelizumab plus chemotherapy in locally advanced ESCC were identified through literature search. Raw data from investigators were pooled for analysis. Primary endpoint was pCR; secondary endpoints included MPR, R0 resection rate, EFS, DFS, and OS.

**Results:**

Six studies involving 306 patients were analyzed; 275 (89.9%) underwent surgery. Among surgical patients, pCR rate was 25.5% and MPR rate was 49.5%. Most patients (98.5%) achieved R0 resection. At median follow-up of 31.3 months, median EFS, DFS, and OS were not reached. The 1-/2-/3-year rates were: EFS 81.8%/66.9%/59.1%; DFS 79.0%/68.7%/62.3%; and OS 91.7%/77.5%/73.4%. Tislelizumab dose intensity correlated with MPR. MPR and R0 resection were independent prognostic factors for EFS and OS, while pathological staging was associated with DFS and OS.

**Conclusions:**

Neoadjuvant tislelizumab combined with chemotherapy demonstrated promising efficacy with encouraging pathological responses and survival outcomes in locally advanced ESCC, supporting clinical application.

**Supplementary Information:**

The online version contains supplementary material available at 10.1007/s00262-025-04240-8.

## Background

Esophageal squamous cell carcinoma (ESCC) is most prevalent across Eastern to Central Asia, particularly in China, where it accounts for approximately 90% of esophageal cancer cases [1, 2]. Most ESCC patients present with advanced disease at diagnosis, resulting in a poor prognosis and a 5-year survival rate of only around 30% for those undergoing surgery alone [3, 4]. The landmark studies, CROSS and NEOCRTEC5010, have demonstrated that neoadjuvant chemoradiotherapy represents an effective treatment strategy for locally advanced ESCC [4, 5]. Additionally, neoadjuvant chemotherapy has been more commonly employed in the Chinese population, with regimens predominantly consisting of paclitaxel in combination with platinum-based agents [6–8]. However, the long-term survival remains disappointingly low due to frequent recurrence, which continues to pose a significant challenge in this population [6]. Therefore, the clinical needs for effective ESCC management remain unmet.

Programmed cell death 1 (PD-1) inhibition has demonstrated a significant and prolonged survival benefit for patients with advanced ESCC in numerous clinical studies, such as KEYNOTE-590, KEYNOTE-181, and Checkmate-648 [9–11]. Tislelizumab has been approved in China and by the FDA for first-line (PD-L1 ≥ 1) and second-line indications in advanced ESCC, based on the RATIONALE-306 trial and the RATIONALE-302 trial, supporting its superior median overall survival (OS) in the first-line and second-line settings, respectively [12, 13]. The encouraging effect in advanced patients provided a theoretical basis for the expansion of immunotherapy to neoadjuvant treatment.

In recent years, an increasing number of studies have investigated the efficacy of immunotherapy-based neoadjuvant strategies for ESCC; [14–16] however, most remain limited by small sample sizes, single-arm design, or lack of long-term survival data [17, 18]. To date, only one randomized controlled phase III trial (ESCORT-NEO/NCCES01) has shown that the combination of PD-1 inhibitor (camrelizumab) and chemotherapy yields a superior pathological complete response (pCR) rate versus chemotherapy alone [19]. The long-term follow-up data for this trial remain immature. Another ongoing phase III trial (NCT04848753) explores the role of neoadjuvant toripalimab plus chemotherapy in ESCC, and data are not yet available. Thus, the current understanding of this neoadjuvant combination therapy for ESCC remains limited by the lack of robust clinical study evidence. In this study, we performed a pooled analysis of studies involving locally advanced ESCC treated with tislelizumab plus chemotherapy as neoadjuvant treatment, with the aim of providing additional support for the evidence regarding the efficacy of this regimen in a large patient population with long-term follow-up.

## Methods

### Search strategy and study selection

We conducted a systematic literature search using the PubMed and Embase databases to identify studies that were published and reported before November 1, 2024. The detailed search strategy is provided in Table S1. The criteria for the selection of the studies that contributed data to this pooled analysis were as follows: prospective or retrospective clinical studies that have been published in journals or conferences; included patients with locally advanced resectable ESCC (stage T1-2N + M0 or T3-4NanyM0); all studies involved patients who received neoadjuvant tislelizumab in combination with chemotherapy; the endpoint must include pCR; and published in English and investigators were willing to provide raw data. The eligibility of the potential articles was initially assessed by reviewing the abstracts. When necessary, the full text was evaluated to determine inclusion. All studies that met the inclusion criteria were included, regardless of whether a complete manuscript was available.

### Procedures

Two investigators independently extracted data from each study using a structured sheet and entered it into an Excel database. The following data were recorded: type of article, clinical trial phase, main inclusion criteria, intervention model, neoadjuvant therapy regimen, number of patients enrolled and undergoing surgery, patient baseline characteristics (e.g., gender, age, Eastern Cooperative Oncology Group [ECOG] performance status, tumor location, and clinical stage), pCR, major pathological response (MPR), R0 surgical resection, pretreatment TNM staging, ypTNM staging, adjuvant therapy, follow-up for survival, and the time of disease progression or death. Each included study was reviewed several times to ensure that the data were neither incorrectly flagged nor missing.

The standard surgical approach consisted of a two-field lymphadenectomy (thoracic and abdominal fields) or an extended two-field lymphadenectomy. Cervical lymph node dissection (three-field lymphadenectomy) was selectively performed in patients with suspected positive cervical lymph nodes detected on post-neoadjuvant imaging assessment, or in those with upper thoracic esophageal tumors who had positive recurrent laryngeal nerve lymph nodes confirmed by intraoperative frozen section analysis.

### Outcomes

The primary endpoint was pCR of the surgery population, defined as the complete absence of intact tumor cells in the resected specimen. The secondary endpoints included MPR of the surgery population, and the pCR, MPR, downstaging, R0 surgical resection, event-free survival (EFS), disease-free survival (DFS), and OS of the overall pooled population. MPR was defined as the ≤ 10% viable tumor cells in the resected primary tumor and all resected lymph nodes. R0 surgical resection was defined as no residual disease at surgical margins. Downstaging was defined as a reduction in pathologic staging after neoadjuvant therapy compared with baseline. EFS was calculated from the initiation of the neoadjuvant treatment to the date of the following events, whichever came first: any progression of disease precluding surgery, progression or relapse after surgery, or death from any cause. DFS was defined as the time from the surgery to disease progression, relapse, or death, whichever came first. OS was defined as the time from the initiation of the neoadjuvant treatment until death.

### Statistical analysis

The efficacy was evaluated within the overall pooled population and the surgery population after pooling the results of all studies. The overall pooled population included all patients who received at least one dose of tislelizumab plus chemotherapy. The surgery population included all patients who underwent surgery after neoadjuvant treatment. Categorical variables were summarized as percentages while continuous variables as median and interquartile range as warranted. For pCR and MPR, hazard ratios (HR) and associated 95% confidence intervals (CIs) were assessed with the Clopper–Pearson method. EFS, DFS, and OS were estimated with the Kaplan–Meier method, and the associated 95% CIs were evaluated based on Greenwood's formula. We conducted a subgroup analysis of MPR, EFS, DFS, and OS based on the combined individual patient data, aiming to investigate potential prognostic factors. The subgroup of MPR was evaluated by univariate analysis and multivariate logistic regression analysis, and survival was Cox regression analysis. A prespecified “full model” strategy: all clinically relevant baseline and pathological covariates were entered into the multivariate models irrespective of univariable significance. Bayesian information criterion (BIC)-guided stepwise Cox regression is a sensitivity analysis to address model parsimony and potential overfitting. Propensity-score matched (PSM) was used to control for the confounders between the adjuvant therapy group and the non-adjuvant therapy group. Propensity scores (PS) were calculated through logistic regression modeling with variables including age, sex, ECOG performance status, dose intensity of tislelizumab and taxane, resection, ypT stage, ypN stage, and MPR. A 1:1 ratio math was performed on the estimated PS using the greedy nearest neighbor matching algorithm. Covariate balance was evaluated using standardized differences; small absolute values (< 0.1) indicated balance. Statistical analyses were performed with R (version 4.2.3). Tests were two-sided, with *p* < 0.05 considered statistically significant.

## Results

### Patient characteristics

Six studies were included in the final pooled analysis, five of which were identified through database searches. The remaining study referred to immunotherapy without specifying the agent; however, correspondence with the author confirmed that tislelizumab was administered. Standardized characteristics of the included clinical trials are listed in Table 1. Overall, the studies included two open-label, single-arm phase II trials, one randomized dual-cohort phase II trial, and three retrospective studies. A total of 306 patients with locally advanced resectable ESCC were treated with neoadjuvant tislelizumab plus chemotherapy, who were available for analysis; eight patients were excluded due to having received other immunotherapy or incomplete data. Tislelizumab was administered intravenously on day 1 of a 21-day cycle in each study. MPR and pCR were the most frequently used primary endpoints (*n* = 3 each), with EFS selected in one study.

**Table 1:** Summary of the studies

Reference	Trial identifier	Study design	Study population	No. of patients received tislelizumab (overall/surgery population)	Tislelizumab (dose)	Chemotherapy regimen	Primary endpoint
Yao, et al. [20]	NCT05807542	Phase II	T2-4N0-1M0	18/15	200 mg Q3W	Carboplatin + nab-PTX	pCR
Wang, et al. [21, 22]	ChiCTR2300073320	Phase II	T1-2N1-2M0 or T3-4aN0-3 M0	46/42^*^	200 mg Q3W	Cisplatin + nab-PTX or PTX	MPR
Yan, et al. [17]	ChiCTR2000037488	Phase II	T2-4aNxM0	45/36	200 mg Q3W	Carboplatin + nab-PTX	MPR
Wang, et al. [23]	SQ2022–116	Retrospective	T3-4aN0-3M0 or M1	122/99	200 mg Q3W	Cisplatin + PTX or nab-PTX	pCR and MPR
Liao, et al. [24, 25]	Med-Eth-Re (2024) 253	Retrospective	Stage II-IVA	34/34	200 mg Q3W	Taxanes + platinum-based drug	EFS
Yang, et al. [26, 27]	B2022-271R	Retrospective	T2N1-3M0, T3N0-3M0, or T4aN0-2M0	53/53^†^	200 mg Q3W	Taxanes + platinum-based drug	pCR

*Forty-two patients who completed neoadjuvant therapy and underwent surgery from this study were included in the pooled analysis

†The published study reported outcomes of 40 patients who received neoadjuvant tislelizumab and chemotherapy, analyzed after PSM. Raw data from 53 patients before PSM were obtained and included in the pooled analysis

*ESCC* Eastern cooperative oncology group, *Q3W* Once every 3 weeks, *nab-PTX* Albumin-bound paclitaxel, *PTX* Paclitaxel, *pCR* Pathological complete response, *MPR* Major pathological response, *EFS* Event-free survival, *PSM* Propensity-score matched

The baseline characteristics and relative treatment information of the included patients are summarized in Table 2. In the overall pooled population, the median age was 61 years (range, 33–81 years). The majority of patients were male (79.7%), had an ECOG performance status of 0 (84.3%), T3 tumors (73.5%), and were at stage III (70.9%). Most patients (99.3%) completed more than two cycles of neoadjuvant treatment, of which 23 (7.5%) completed treatment for ≥ 4 cycles, respectively. The chemotherapy administered during the neoadjuvant period consisted of a combination of taxane and platinum. Taxane included nab-paclitaxel (nab-PTX; 75.5%) and paclitaxel (PTX; 24.5%), and platinum included cisplatin (47.7%), carboplatin (27.8%), lobaplatin (16.0%), nedaplatin (7.5%), and oxaliplatin (1.0%). The median dose intensity of tislelizumab was 0.67 (range, 0.24–1.5; Table S2).

**Table 2:** Patient characteristics and treatment information in the overall pooled population

Characteristics	Overall pooled population (*n* = 306)
Age, median (range)	61 (33–81)
Sex, male	244 (79.7)
BMI, kg/m^2^	21.9 (15.6–33.3)
*ECOG performance status*	
0	258 (84.3)
1	48 (15.7)
*cT stage*	
1	3 (1.0)
2	46 (15.2)
3	222 (73.5)
4	31 (10.3)
*cN stage*	
0	40 (13.1)
1	121 (39.5)
2	127 (41.5)
3	11 (3.6)
Unknown	7 (2.3)
*AJCC clinical stage*	
I	1 (0.3)
II	55 (18.2)
III	214 (70.9)
IVA	32 (10.6)
*Tumor location*	
Upper	47 (18.0)
Middle	141 (54.0)
Lower	73 (28.0)
*Neoadjuvant cycles*	
1	2 (0.7)
2	203 (66.3)
3	78 (25.5)
4	20 (6.5)
5	3 (1.0)
Dose intensity of tislelizumab, median (range)	0.67 (0.24–1.50)
Dose intensity of taxane, median (range)	0.69 (0.14–1.52)
*Taxane*	
Paclitaxel	75 (24.5)
Nab-paclitaxel	231 (75.5)
*Platinum*	
Cisplatin	146 (47.7)
Carboplatin	85 (27.8)
Nedaplatin	23 (7.5)
Lobaplatin	49 (16.0)
Oxaliplatin	3 (1.0)

Data are presented as median (range) or *n* (%)

*BMI* Body mass index, *ECOG* Eastern cooperative oncology group, *AJCC* American joint committee on cancer

### Surgery and survival

Out of 306 patients, 275 underwent surgery, and all performed using a minimally invasive approach (Table 3). The median interval between the end of neoadjuvant and surgery was 33 days (IQR, 28–40). The remaining patient who had surgery canceled due to various reasons, including refusal (*n* = 18), adverse event (AE; *n* = 5), disease progression or recurrence (*n* = 5), and other reasons (*n* = 3). Of patients who underwent surgery, the majority reached R0 resection, with an R0 resection rate of 98.5%. A median of 29 (IQR, 19–40) lymph nodes were dissected during surgery. The pCR rate was reported at 25.5% (95% CI 20.4–31.1), and the MPR rate reached 49.5% (95% CI 43.5–55.5) in the surgery population. Among the overall pooled population, the pCR rate and MPR rate were 22.9% (95% CI 18.3–28.0) and 44.4% (95% CI 38.9–50.1), respectively. Downstaging of the T stage was observed in 168 (61.1%) patients, as well as N stage downstaging occurred in 180 (65.5%) patients.

**Table 3:** Surgery and pathological outcomes

Characteristics	Surgery population (*n* = 275)
Time to surgery (days), median (IQR)	33 (28–40)
Number of lymph node dissections, median (IQR)	29 (19–40)
*Resection*	
R0	271 (98.5)
R1	1 (0.04)
R2	3 (1.1)
*ypT stage*	
T0	75 (27.3)
T1	55 (20.0)
T2	51 (18.5)
T3	73 (26.5)
T4	19 (6.9)
Unknown	2 (0.7)
*ypN stage*	
N0	164 (59.6)
N1	68 (24.7)
N2	33 (12.0)
N3	8 (2.9)
Unknown	2 (0.7)
*Adjuvant therapy*	
Yes	138 (50.2)
No	137 (48.8)
Time to adjuvant therapy (days), median (IQR)	51 (43–61)
*Pathological response*	
pCR	70 (25.5, 20.4–31.1)
MPR	136 (49.5, 43.5–55.5)

Data are presented as median (IQR), *n* (%), or *n* (%, 95% CI).

*ypT* Post-neoadjuvant therapy pathological Tumor, *ypN* Post-neoadjuvant therapy pathological Nodes, *pCR* Pathological complete response, *MPR* Major pathological response, *CI* confidence intervals

With a median follow-up of 31.3 months, the median EFS, median OS, and median DFS were not reached in the overall pooled population. The 1-year EFS rate was 81.8% (95% CI 77.6–86.3), the 2-year rate was 66.9% (95% CI 61.5–72.7), and the 3-year rate was 59.1% (95% CI 53.1–65.7). The 1-year, 2-year, and 3-year OS rates were 91.7% (95% CI 88.7–94.9), 77.5% (95% CI 72.5–82.8), and 73.4% (95% CI 67.8–79.3), respectively. The 1-year, 2-year, and 3-year DFS rates were 79.0% (95% CI 74.3–84.1), 68.7% (95% CI 63.0–74.9), and 62.3% (95% CI 56.0–69.2; Figure S1).

### Prognostic factors of pathological response and long-term survival

We further evaluated the potential prognostic factors associated with pathological response and long-term survival in patients treated with neoadjuvant tislelizumab in combination with chemotherapy. Regarding the pathological response, logistic regression analysis showed that the dose intensity of tislelizumab (*p* = 0.031) was an independent predictor of MPR (Table S3).

**Fig. 1:**
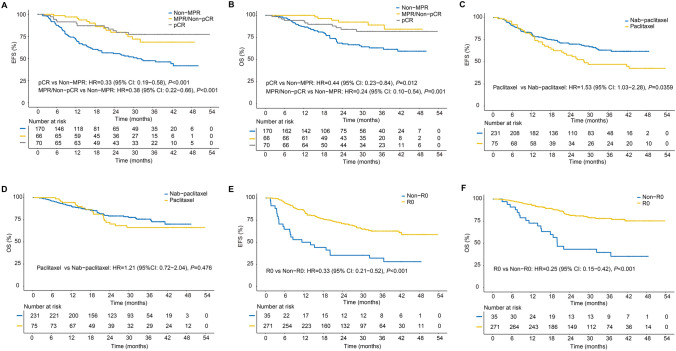
Comparison of EFS and OS among patients with varying factors. **A**–**B** Comparison of EFS and OS among patients with pCR and MPR; **C**–**D** comparison of EFS and OS among patients who received paclitaxel and nab-paclitaxel; and **E**–**F** comparison of EFS and OS among patients who reached R0 resection and non-R0 resection

Kaplan–Meier analysis revealed that patients who achieved pCR and MPR showed a comparable trend and had a significantly better EFS and OS than those with other patients (all *p* < 0.05; Fig. 1A–B). Patients who reached R0 resection had a more significant EFS and OS benefit than others (all *p* < 0.001), whereas patients treated with PTX demonstrated a significantly worse EFS than those treated with nab-PTX (*p* = 0.0359), but this was not significant in OS (*p* = 0.476; Fig. 1C–F). The clinical stage, the dose intensity of tislelizumab, and chemotherapy were not significantly associated with EFS and OS (Figure S2). In the multivariate analyses, only the cN2/3 stage, MPR, and R0 resection retained their prognostic value (all *p* < 0.05; Table 4). These findings were further corroborated by the BIC-selected model (Table S4).

**Table 4:** Univariate and multivariate Cox regression for EFS and OS

	EFS				OS			
	Univariate analysis		Multivariate analyses		Univariate analysis		Multivariate analyses	
	HR (95% CI)	P^*^	HR (95% CI)	P^*^	HR (95% CI)	P^*^	HR (95% CI)	P^*^
Age, > 65	0.93 (0.63–1.37)	0.697	1.00 (0.66–1.52)	0.992	0.91 (0.56–1.50)	0.720	1.15 (0.67–1.95)	0.616
Sex, male	1.21 (0.76–1.93)	0.423	1.33 (0.8–2.19)	0.269	1.12 (0.62–2.01)	0.709	1.31 (0.69–2.47)	0.412
ECOG of 1	0.99 (0.59–1.69)	0.985	0.77 (0.44–1.35)	0.365	0.85 (0.42–1.71)	0.651	0.61 (0.29–1.27)	0.183
cT ¾	1.24 (0.74–2.09)	0.408	1.43 (0.84–2.46)	0.191	1.22 (0.64–2.33)	0.546	1.51 (0.76–3)	0.243
cN 2/3	1.71 (1.17–2.50)	**0.005**	1.56 (1.03–2.37)	**0.035**	1.84 (1.14–2.99)	**0.013**	1.95 (1.13–3.37)	**0.016**
IM dose, high dose	0.78 (0.51–1.20)	0.257	1.23 (0.69–2.18)	0.483	1.05 (0.63–1.76)	0.841	1.76 (0.86–3.59)	0.122
PTX dose, high dose	0.90 (0.62–1.32)	0.588	0.96 (0.6–1.55)	0.876	1.15 (0.72–1.86)	0.557	1.12 (0.61–2.06)	0.710
PTX	1.54 (1.03–2.28)	0.0347	1.41 (0.9–2.22)	0.138	1.21 (0.72–2.04)	0.476	1.3 (0.72–2.35)	0.387
MPR	0.35 (0.23–0.54)	** < 0.001**	0.4 (0.25–0.63)	** < 0.001**	0.34 (0.20–0.59)	** < 0.001**	0.4 (0.22–0.74)	**0.004**
R0 resection	0.33 (0.21–0.52)	** < 0.001**	0.46 (0.28–0.76)	**0.003**	0.25 (0.15–0.42)	** < 0.001**	0.36 (0.2–0.65)	**0.001**

*P< 0.05 was considered statistically significant

*EFS* Event-free survival, *OS* Overall survival, *HR* Hazard ratio, *CI* Confidence intervals, *ECOG* Eastern cooperative oncology group, *cT* Clinical stage T, *cN* Clinical stage N, *IM* Immunotherapy, *PTX* Paclitaxel, *MPR* Major pathological response

**Fig. 2:**
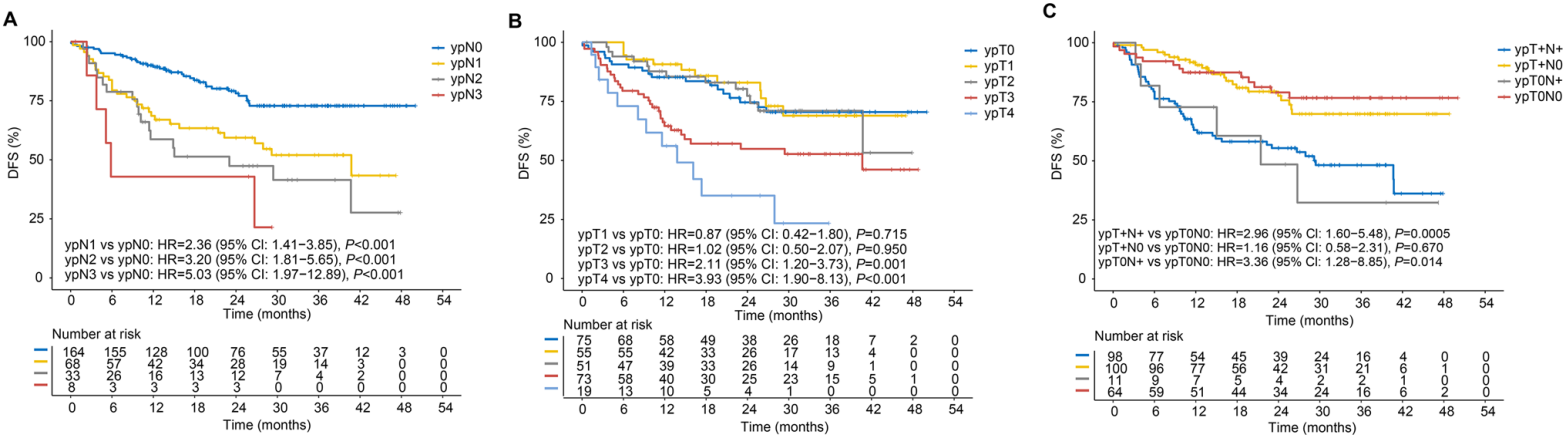
Comparison of DFS among patients with different pathological stages. **A** Comparison of DFS among patients with different ypN stages; **B** comparison of DFS among patients with different ypT stages; and **C** comparison of DFS among patients with ypN + , ypT + , and ypT0N0 stages

We further explored the influence of post-neoadjuvant therapy pathological staging on long-term survival. Univariate analysis demonstrated that ypN1/2/3 stages were significant prognostic factors for poor DFS (all *p* < 0.001), while ypN2/3 stages were associated with reduced OS (all *p* < 0.05; Fig. 2A, Figure S3A). Both DFS and OS benefits were more pronounced in patients with ypT0-2 stages, whereas patients with ypT3/4 stages exhibited a poorer prognosis, particularly those with ypT4 (all *p* < 0.001; Fig. 2B, Figure S3B). Comprehensive evaluation of ypTN staging indicated that ypN had a more substantial impact on DFS compared to ypT (ypT0N + vs. ypT0N0: *p* = 0.014; ypT + N0 vs. ypT0N0: *p* = 0.670; Fig. 2C). However, no significant trend was observed for OS (all *p* > 0.05; Figure S3C).

**Fig. 3:**
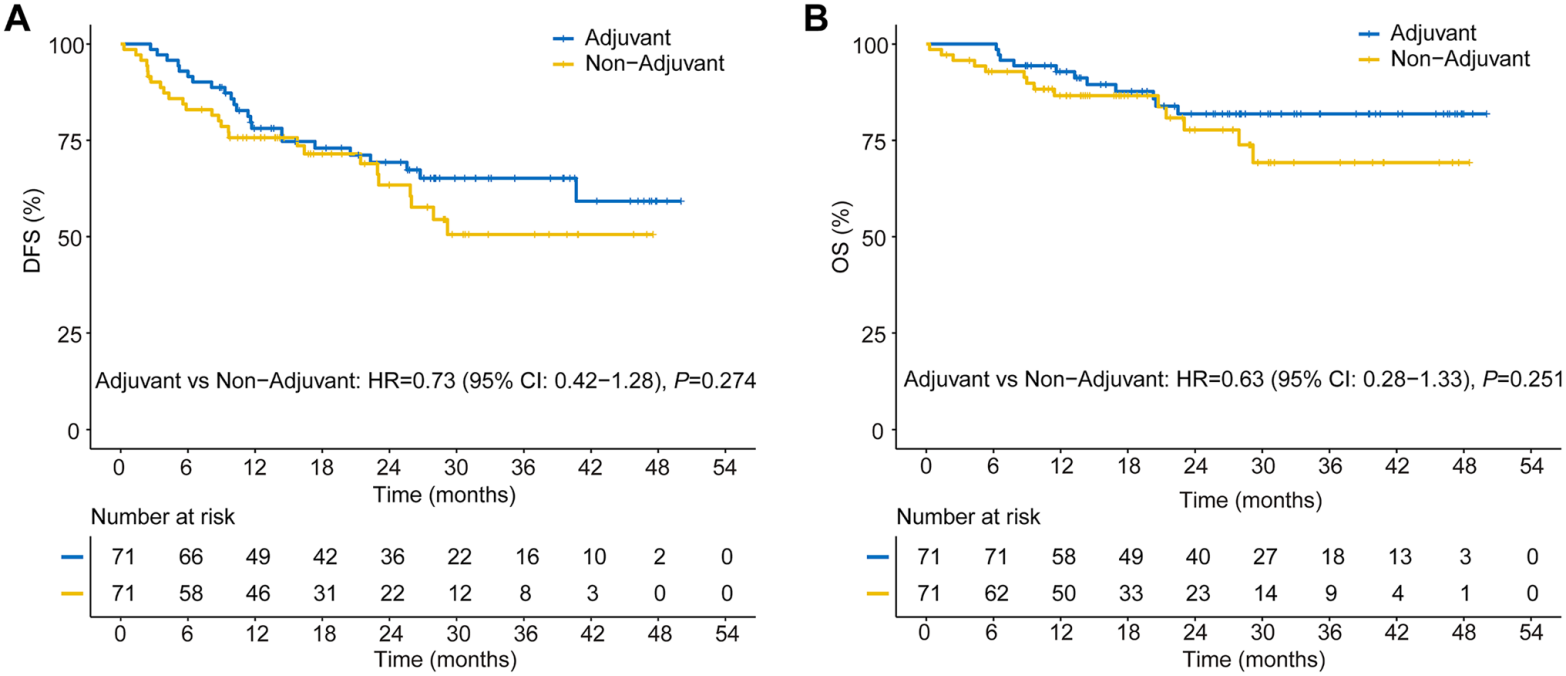
Comparison of DFS and OS among the adjuvant therapy group and the non-adjuvant therapy group after propensity-score matched. **A** DFS in patients with or without adjuvant therapy; **B** OS in patients with or without adjuvant therapy

We further compared the DFS and OS between patients receiving adjuvant therapy and those who did not. To better control for the treatment bias, PSM of the patients was matched 1:1 ratio. The resulting PSM cohort included 106 patients, yielding 71 and 71 patients in the adjuvant and non-adjuvant therapy groups, respectively. After PSM, the baseline characteristics were comparable in the two groups (Table S5). There was no significant survival advantage for patients who received adjuvant therapy versus those who did not, but a trend toward improved DFS and OS was observed in the adjuvant therapy group, as reflected by the Kaplan–Meier curves (Fig. 3). Moreover, patients were categorized into subgroups based on their pathological response status, and the proportion receiving adjuvant therapy was substantially lower in those who achieved pCR or MPR compared to the non-pCR or non-MPR subgroups (all *p* < 0.05; Table S6). We further examined DFS according to adjuvant therapy status within each pathological response stratum. Among patients with MPR, adjuvant therapy was not associated with improved DFS (*p* = 0.846). In contrast, among patients without MPR, adjuvant therapy showed a trend toward better DFS, although this did not reach statistical significance (*p* = 0.188).

## Discussion

The current studies evaluating the efficacy of neoadjuvant immunotherapy combined with chemotherapy in patients with locally advanced ESCC are limited to small sample sizes and single-arm or retrospective study designs, with no randomized phase III trials yet reporting long-term outcomes [18, 28]. To our knowledge, this is the first pooled analysis of individual patient data to evaluate the efficacy of the neoadjuvant tislelizumab plus chemotherapy, showing that 25.5% of surgery patients achieved pCR and 49.5% achieved MPR, with encouraging long-term survival outcomes also observed. Moreover, we also explored the independent predictors for pathological response and long-term survival. We believe these findings provide evidence to support future larger clinical trials and the application of neoadjuvant immunochemotherapy in ESCC.

In recent years, piles of small-sample-size studies have investigated the preliminary efficacy of PD-1 inhibitors plus chemotherapy in locally advanced ESCC, with pCR ranging from 21.7 to 50.0% and MPR from 44.4 to 72.0% [17, 19, 29–32]. Our pooled analysis of 306 patients confirmed and extended the findings from prior studies, showing that tislelizumab-containing regimens yield comparable pathological response (pCR, 25.5%; MPR, 49.5%). Moreover, the comparable benefit observed in our study with the ESCORT-NEO/NCCES01 study (pCR, 28.0% in nab-PTX arm) may further support the superior efficacy of tislelizumab in locally advanced ESCC [17–19], particularly since the suboptimal dose intensity of chemotherapy in some patients in our study could have influenced the observed outcomes [23], although direct cross-trial comparisons cannot be made.

The most striking finding of this pooled analysis was the provision of a comprehensive report on long-term survival, particularly noteworthy given the lack of such data in other studies. Although a limitation of the pooled survival analysis is the variation in follow-up times across studies, the observed survival trends suggested long-term clinical benefit with this regimen, aligning with prior similar reports (1-year OS rate, 91.7% vs. 86.7–90.9%; 2-year OS rate, 77.5% vs. 75.6–81.3%) and numerically higher than historical outcomes with doublet chemotherapy or doublet chemoradiotherapy alone (3-year OS rate, 73.4% vs. 51.9–65.8%) [6, 18, 32–35]. The recent JCOG1109 study demonstrated that triplet chemotherapy significantly improved OS compared with doublet chemotherapy (3-year OS rate, 72.1% vs. 62.6%; HR 0.68 [95% CI 0.50–0.92]; *p* = 0.006) [36]. Encouragingly, the 3-year OS rate (73.4%) observed in our study with tislelizumab plus chemotherapy appears comparable to the triplet chemotherapy of JCOG1109. However, well-designed randomized controlled trials are needed in the future to directly compare the regimens. The sustained long-term benefits of our study were further evidenced by the shape of the OS curve, which showed an apparent plateau beginning at approximately 24 months. The cross-trial comparisons, however, need to be interpreted with caution due to the heterogeneity in treatment regimen, design, and prognosis characteristics.

The univariate and multivariate analyses identified several potential prognostic factors. Firstly, patients who received high-dose intensity of tislelizumab were associated with improved MPR, highlighting the importance of maintaining optimal dose intensity, ensuring timely administration of adequate doses. Secondly, several studies have demonstrated that MPR was correlated with enhanced survival in solid tumors [37–39]. Our results suggest that patients who achieved either MPR or pCR showed comparable EFS and OS, indicating that a wider population may derive therapeutic benefits from this regimen, even without achieving pCR, providing support for MPR as an early endpoint for survival in similar studies. Moreover, patients who received nab-PTX treatment also exhibited a favorable EFS than those who used PTX, potentially due to its unique advantages and superior safety [40, 41], whereas it was not replicated in terms of OS. Additionally, the prognostic value of the ypN stage was more pronounced than that of the ypT stage, particularly in patients with ypN2/3 disease. This was not unexpected, as these patients generally exhibited poor prognostic features, and improved survival outcomes were typically associated with complete or subtotal lymph node regression [42–44]. These findings further highlight the importance of nodal status in reflecting residual micrometastatic burden and the efficacy of systemic disease control, echoing the rationale for extended lymphadenectomy in early-stage disease observed in the JCOG0502 study [36].

Adjuvant immunotherapy is expected to prolong survival for EC patients; to date, only the CheckMate 577 study has demonstrated positive outcomes (median DFS: nivolumab vs. placebo, 21.8 vs. 10.8 months; HR = 0.76 [95% CI 0.63–0.91]) in patients who had received neoadjuvant chemoradiotherapy without achieving pCR [45, 46]. However, given the current widespread use of neoadjuvant immunotherapy in clinical practice, the value of these findings was limited by the inclusion of patients who received neoadjuvant chemotherapy. Moreover, ongoing investigations into the benefit of adjuvant therapy have yet to yield conclusive results. Our study revealed an encouraging trend in both DFS and OS within the adjuvant treatment cohort, during the early stage, which persisted throughout the observation period. Notably, among patients without MPR, adjuvant immunotherapy showed a consistent trend toward improved outcomes, aligning with the findings of the CheckMate 577 study [45]. Although the absence of statistical significance, it suggests that adjuvant immunotherapy may hold potential benefit, particularly for patients who fail to achieve MPR after neoadjuvant therapy. Considering the heterogeneity in adjuvant regimens across studies, including variations in drug selection and treatment cycles, as well as potential investigator-driven biases in determining eligibility for adjuvant therapy, these findings should be interpreted cautiously. Additionally, to address the key limitation of adjuvant therapy with the inability to definitively identify patients at high risk and the lack of an optimal regimen, further trials are warranted to evaluate personalized and risk-adapted treatment strategies, potentially integrating biomarkers (e.g., minimal residual disease, circulating tumor DNA) to enhance therapeutic precision [47].

The main limitation of this pooled analysis was that the included studies were non-randomized, with half being retrospective, which weakened the strength of our findings and may have introduced bias into conclusions. Another limitation of this pooled analysis is the lack of integrated safety data. Because of heterogeneity in AE reporting and incomplete availability of AE and immune-related AE data across the included studies, a pooled safety analysis of all six studies was deemed inappropriate. We have summarized the results from the four studies with complete AE data in Table S7; however, given the incomplete reporting, these findings should be interpreted with caution. The safety profile should be comprehensively assessed in further research. Although we explored the potential prognostic factors associated with pathological response and long-term survival, the imbalance in baseline characteristics across patients led to smaller sample sizes in some groups, potentially compromising the robustness of the statistical findings. Additionally, our PSM analysis should also consider several limitations: First, while we balanced all available clinical and pathological variables, residual confounding from unmeasured factors (such as specific tumor biological characteristics or detailed surgical parameters) may persist. Second, the inclusion of patients from multiple trials introduces inherent heterogeneity in treatment protocols, patient selection, and follow-up procedures. Third, the matching process reduced our sample size, potentially limiting statistical power to detect smaller effect sizes. Despite these limitations, the directional trend toward improved survival outcomes with adjuvant therapy provides valuable insights for future randomized studies.

In conclusion, this pooled analysis showed promising clinical outcomes for tislelizumab combined with chemotherapy as a neoadjuvant treatment in a large cohort population, providing clinical evidence to support the wide application of this treatment option. The findings of this study serve as a basis for future research, and long-term, large-scale clinical trials are warranted to validate the benefits of this treatment regimen and its prognostic implications. Furthermore, as neoadjuvant immunotherapy plus chemoradiotherapy gains momentum, future studies are also likely to focus on elucidating the relative benefits of immunotherapy plus concurrent versus sequential chemoradiotherapy, paving the way for optimized neoadjuvant strategies.

## Supplementary Information

Below is the link to the electronic supplementary material.

Supplementary file1 (DOCX 468801 KB)

## Data Availability

All data relevant to the study are included in the article or uploaded as supplementary information.

## Abbreviations

ESCC: Esophageal squamous cell carcinoma
PD-1: Programmed cell death 1
OS: Overall survival
pCR: Pathological complete response
MPR: Major pathological response
EFS: Event-free survival
DFS: Disease-free survival
HR: Hazard ratios
Cis: Confidence intervals
PSM: Propensity-score matched
PS: Propensity scores
