# Supplementary material for: Long-term outcome of neoadjuvant tislelizumab plus chemotherapy in locally advanced esophageal squamous cell carcinoma
Source: Cancer Immunol Immunother. 2025 Dec 18;75(1):2. doi: 10.1007/s00262-025-04240-8 (PMC12715071; doi:10.1007/s00262-025-04240-8)
Supplement: Supplementary file 1 — Supplementary file1 (DOCX 468801 KB) [file 262_2025_4240_MOESM1_ESM.docx]

# **Supplementary Table**

**Table S1. The detailed strategies in the two databases**

| **Databases** | **Search Strategies** |
| --- | --- |
| PubMed (-November 1, 2024. 12 articles) | ((("esophageal squamous cell carcinoma" OR esophageal squamous carcinoma OR squamous cell carcinoma of the esophagus OR oesophageal squamous cell carcinoma OR esophageal squamous cell cancer OR ESCC OR "esophageal squamous cell carcinoma"[MeSH]) AND (neoadjuvant OR "neo-adjuvant" OR preoperative OR "pre-operative" OR preoperation OR preoperations OR "pre-operation" OR "pre-operations" OR presurgical OR "pre-surgical" OR presurgery OR "pre-surgery" OR preresection OR "pre-resection" OR preresectional OR "before surgery" OR "before resection" OR "before operation" OR "prior to resection" OR "prior to surgery" OR "prior to operation" OR "neoadjuvant therapy"[MeSH])) AND (tislelizumab OR "BGB-A317")) AND (chemotherapy OR "chemical therapy" OR oxaliplatin OR fluorouracil OR "5-FU" OR "5-fluoruracil" OR "5-fluorouracil" OR docetaxel OR cisplatin OR capecitabine OR paclitaxel OR "paclitaxel liposome" OR "albumin-bound paclitaxel" OR "nab paclitaxel" OR irinotecan OR vinorelbine OR "Tegafur Gimeracil Oteracil" OR "TS-1" OR "S-1" OR nedaplatin OR oxaliplatin[MeSH] OR fluorouracil[MeSH] OR docetaxel[MeSH] OR cisplatin[MeSH] OR capecitabine[MeSH] OR paclitaxel[MeSH] OR "albumin-bound paclitaxel"[MeSH] OR irinotecan[MeSH] OR vinorelbine[MeSH]) |
| Embase (-November 1, 2024. 77 entries) | ('esophageal squamous cell carcinoma'/exp OR 'esophageal squamous cell carcinoma' OR 'esophageal squamous carcinoma' OR 'squamous cell carcinoma of the esophagus'/exp OR 'squamous cell carcinoma of the esophagus' OR 'oesophageal squamous cell carcinoma'/exp OR 'oesophageal squamous cell carcinoma' OR 'esophageal squamous cell cancer'/exp OR 'esophageal squamous cell cancer' OR escc) AND (neoadjuvant OR 'neo-adjuvant' OR preoperative OR 'pre-operative' OR preoperation OR preoperations OR 'pre-operation' OR 'pre-operations' OR presurgical OR 'pre-surgical' OR presurgery OR 'pre-surgery' OR preresection OR 'pre-resection' OR preresectional OR 'before surgery' OR 'before resection' OR 'before operation' OR 'prior to resection' OR 'prior to surgery' OR 'prior to operation') AND ('tislelizumab'/exp OR tislelizumab) AND ('chemotherapy'/exp OR chemotherapy OR 'chemical therapy' OR 'oxaliplatin'/exp OR oxaliplatin OR 'fluorouracil'/exp OR fluorouracil OR '5-fu'/exp OR '5-fu' OR '5-fluoruracil'/exp OR '5-fluoruracil' OR '5-fluorouracil'/exp OR '5-fluorouracil' OR 'docetaxel'/exp OR docetaxel OR 'cisplatin'/exp OR cisplatin OR 'capecitabine'/exp OR capecitabine OR 'paclitaxel'/exp OR paclitaxel OR 'paclitaxel liposome'/exp OR 'paclitaxel liposome' OR 'albumin-bound paclitaxel'/exp OR 'albumin-bound paclitaxel' OR 'nab paclitaxel'/exp OR 'nab paclitaxel' OR 'irinotecan'/exp OR irinotecan OR 'vinorelbine'/exp OR vinorelbine OR 'tegafur gimeracil oteracil'/exp OR 'tegafur gimeracil oteracil' OR 'ts-1'/exp OR 'ts-1' OR 's-1'/exp OR 's-1' OR 'nedaplatin'/exp OR nedaplatin) |

**Table S2. Dose intensity of chemotherapy and tislelizumab**

| **Chemotherapy** | Dose intensity = Actual dose intensity/standard dose intensity×100%  Actual dose intensity (mg/m^2^/cycle):  Cycle ≥3: 21×(sum of actual drug doses in each cycle)/(date of last dose–date of first dose + 21)  Cycle <3: 21×Sum of actual medication doses for each cycle / [(Last medication date + 21 + Remaining cycles×21) - First medication date], where Remaining cycles = 3 - Actual medication cycles  Plan dose intensity:  Paclitaxel: 135mg/m^2^  Nab-paclitaxel: 125mg/m^2^  Cisplatin: 75mg/m^2^  Nedaplatin: 80mg/m^2^×body surface area/21 (the instruction dose: 80-100mg/m^2^)  Carboplatin: AUC = 5 (the patient's creatinine needs to be known)  Lobaplatin: 50mg/m^2^ (the instruction dose) |
| --- | --- |
| **Tislelizumab** | Actual dose intensity (mg/cycle):  Cycle ≥3: 21×(sum of actual drug doses in each cycle)/(date of last dose–date of first dose + 21)  Cycle <3: 21×Sum of actual medication doses for each cycle / [(Last medication date + 21 + Remaining cycles×21) - First medication date], where Remaining cycles = 3 - Actual medication cycles  Plan dose intensity (mg/cycle) = planned dose per cycle (mg), 200mg.  Relative dose intensity = actual dose intensity/200mg×100%. |

**Table S3. Univariable and multivariable Cox regression for MPR**

|  | **Univariate analysis** | | **Multivariate analysis** | |
| --- | --- | --- | --- | --- |
|  | **OR (95% CI)** | ***P* value**^*^ | **OR (95% CI)** | ***P* value**^*^ |
| Age, >65 | 1.02 (0.64-1.62) | 0.937 | 1.03 (0.62-1.70) | 0.903 |
| Sex, male | 0.76 (0.43-1.32) | 0.325 | 0.75 (0.40-1.39) | 0.355 |
| ECOG of 1 | 0.98 (0.52-1.81) | 0.938 | 0.89 (0.46-1.70) | 0.720 |
| AJCC T 3/4 | 0.89 (0.48-1.65) | 0.702 | 0.90(0.47-1.73) | 0.744 |
| AJCC N 2/3 | 0.83 (0.53-1.31) | 0.428 | 0.92(0.56-1.50) | 0.729 |
| IM dose | 2.04 (1.24-3.39) | **0.005** | 2.08 (1.07-4.09) | **0.031** |
| PTX dose | 1.21 (0.77-1.91) | 0.417 | 0.84 (0.46-1.51) | 0.558 |
| PTX | 0.54 (0.31-0.92) | **0.027** | 0.63 (0.33-1.16) | 0.143 |

^*^*P*< 0.05 was considered statistically significant.

Abbreviations: BMI, body mass index; ECOG, Eastern Cooperative Oncology Group; AJCC, American Joint Committee on Cancer; IM, immunotherapy; PTX, paclitaxel; OR, odds ratio; CI, confidence intervals.

**Table S4. Univariate and multivariate Cox regression for EFS and OS (using Bayesian Information Criterion-guided stepwise Cox regression)**

|  | **EFS** | | | | **OS** | | | |
| --- | --- | --- | --- | --- | --- | --- | --- | --- |
|  | **Univariate analysis** | | **Multivariate analyses** | | **Univariate analysis** | | **Multivariate analyses** | |
|  | **HR (95% CI)** | ***P* value**^*^ | **HR (95% CI)** | ***P* value**^*^ | **HR (95% CI)** | ***P* value**^*^ | **HR (95% CI)** | ***P* value**^*^ |
| Age, >65 | 0.93 (0.63-1.37) | 0.697 |  |  | 0.91 (0.56-1.50) | 0.720 |  |  |
| Sex, male | 1.21 (0.76-1.93) | 0.423 |  |  | 1.12 (0.62-2.01) | 0.709 |  |  |
| ECOG of 1 | 0.99 (0.59-1.69) | 0.985 |  |  | 0.85 (0.42-1.71) | 0.651 |  |  |
| cT 3/4 | 1.24 (0.74-2.09) | 0.408 |  |  | 1.22 (0.64-2.33) | 0.546 |  |  |
| cN 2/3 | 1.71 (1.17-2.50) | **0.005** | 1.58 (1.07-2.32) | **0.020** | 1.84 (1.14-2.99) | **0.013** | 1.65 (1.01-2.71) | **0.046** |
| IM dose, high dose | 0.78 (0.51-1.20) | 0.257 |  |  | 1.05 (0.63-1.76) | 0.841 |  |  |
| PTX dose, high dose | 0.90 (0.62-1.32) | 0.588 |  |  | 1.15 (0.72-1.86) | 0.557 |  |  |
| PTX | 1.54 (1.03-2.28) | 0.0347 |  |  | 1.21 (0.72-2.04) | 0.476 |  |  |
| MPR | 0.35 (0.23-0.54) | **<0.001** | 0.39 (0.25-0.62) | **<0.001** | 0.34 (0.20-0.59) | **<0.001** | 0.44 (0.24-0.79) | **0.006** |
| R0 resection | 0.33 (0.21-0.52) | **<0.001** | 0.50 (0.31-0.80) | **0.004** | 0.25 (0.15-0.42) | **<0.001** | 0.38 (0.22-0.65) | **0.001** |

^*^*P*< 0.05 was considered statistically significant.

Abbreviations: EFS, event-free survival; OS, overall survival; HR, hazard ratio; CI, confidence intervals; ECOG, Eastern Cooperative Oncology Group; cT, clinical stage T; cN, clinical stage N; IM, immunotherapy; PTX, paclitaxel; MPR, major pathological response.

**Table S5. Baseline clinical characteristics of patients after propensity-score matched**

| **Characteristics** | **Adjuvant (n=71)** | **Non-Adjuvant (n=71)** | ***P* value** |
| --- | --- | --- | --- |
| Age, median (IQR) | 62.0 (56.0, 68.0） | 61.0 (57.0, 66.5) | 0.652 |
| Sex, male | 60 (84.5) | 59 (83.1) | 1.000 |
| ECOG performance status, 0 | 60 (84.5) | 60 (84.5) | 1.000 |
| Dose intensity of tislelizumab, median (IQR) | 0.67 (0.67, 0.67) | 0.67(0.64, 0.67) | 0.196 |
| Dose intensity of taxane, median (IQR) | 0.67 (0.53, 0.75) | 0.65 (0.53, 0.73) | 0.603 |
| Resection |  |  | 1.000 |
| R0 | 70 (98.6) | 70 (98.6) |  |
| Non-R0 | 1 (1.4) | 1 (1.4) |  |
| ypT stage |  |  | 0.998 |
| T0 | 19 (26.8) | 19 (26.8) |  |
| T1 | 15 (21.1) | 16 (22.5) |  |
| T2 | 9 (12.7) | 9 (12.7) |  |
| T3 | 23 (32.4) | 23 (32.4) |  |
| T4 | 5 (7.0) | 4 (5.6) |  |
| ypN stage |  |  | 0.949 |
| N0 | 41 (57.7) | 41 (57.7) |  |
| N1 | 20 (28.2) | 19 (26.8) |  |
| N2 | 9 (12.7) | 9 (12.7) |  |
| N3 | 1 (1.4) | 2 (2.8) |  |
| MPR | 33 (46.5) | 34 (47.9) | 1.000 |

Data are presented as median (IQR) or n (%).

Abbreviations: ECOG, Eastern Cooperative Oncology Group; ypT, post-neoadjuvant therapy pathological Tumor; ypN, post-neoadjuvant therapy pathological Nodes.

**Table S6. Impact of adjuvant therapy on pCR and MPR**

|  | **Adjuvant** | **Non-adjuvant** | ***P* value**^*^ |
| --- | --- | --- | --- |
| **pCR** | 19 (27.1) | 51 (72.9) | **<0.001** |
| **Non-pCR** | 119 (58.0) | 86 (42.0) |  |
| **MPR** | 56 (41.2) | 80 (58.8) | **0.0031** |
| **Non-MPR** | 82 (59.0) | 57 (41.0) |  |

Data are presented as n (%).

^*^*P*< 0.05 was considered statistically significant.

Abbreviations: pCR, pathological complete response; MPR, major pathological response.

**Table S7. Summary of available treatment-related adverse events**

| **Treatment-related adverse event** | **Any Grade** | **Grade ≥3** |
| --- | --- | --- |
| Any treatment-related adverse event | 152 (85.4) | 29 (16.3) |
| Nausea | 65 (36.5) | 3 (1.7) |
| Anemia | 64 (36.0) | 2 (1.1) |
| Vomiting | 60 (33.7) | 3 (1.7) |
| Leukopenia | 51 (28.7) | 13 (7.3) |
| Alopecia | 49 (27.5) | 0 |
| Neutropenia | 31 (17.4) | 8 (4.5) |
| Fatigue | 29 (16.3) | 1 (0.6) |
| Thrombocytopenia | 27 (15.2) | 3 (1.7) |
| Decreased appetite | 16 (9.0) | 0 |
| RBC count decreased | 13 (7.3) | 0 |
| Rash | 9 (5.1) | 1 (0.6) |
| Cardiotoxicity | 8 (4.5) | 0 |
| Pruritus | 8 (4.5) | 1 (0.6) |
| Thyroid dysfunction | 8 (4.5) | 0 |
| Diarrhea | 7 (3.9) | 0 |
| Hypoadrenocorticism | 6 (3.4) | 0 |
| LDH increased | 6 (3.4) | 0 |
| Pneumonia | 6 (3.4) | 0 |
| ALT/AST increased | 5 (2.8) | 0 |
| Constipation | 5 (2.8) | 0 |
| Fever | 4 (2.2) | 0 |
| Blood bilirubin increased | 3 (1.7) | 0 |
| Hypothyroidism | 3 (1.7) | 0 |
| Lipase increased | 3 (1.7) | 1 (0.6) |
| Oral mucositis | 3 (1.7) | 0 |
| γ-GT increased | 3 (1.7) | 0 |
| Dermatitis | 1 (0.6) | 0 |
| Hyperglycemia | 1 (0.6) | 0 |
| Hyperpotassium | 1 (0.6) | 0 |
| Hypertriglyceridemia | 1 (0.6) | 0 |
| Hypoproteinemia | 1 (0.6) | 0 |
| Peripheral sensory neuropathy | 1 (0.6) | 0 |

Data are presented as n (%).

# **Supplementary Figure**

**Figure S1. Kaplan-Meier curves for EFS, OS, and DFS**

(A) Kaplan-Meier curves of EFS among the overall pooled population; (B) Kaplan-Meier curves of OS among the overall pooled population; (C) Kaplan-Meier curves of DFS among the surgery population.

**
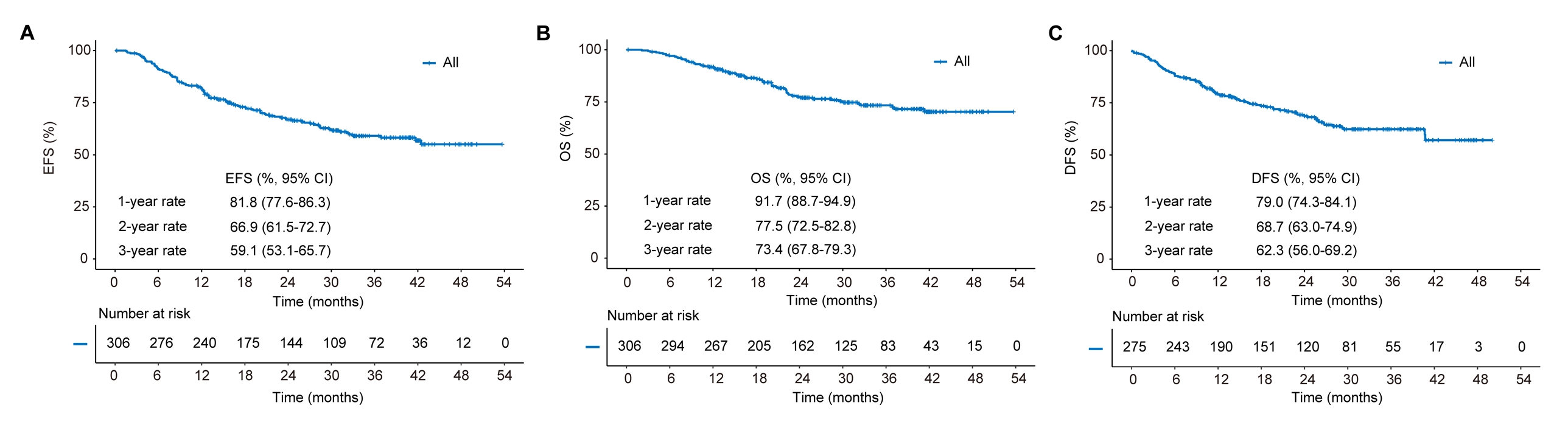
**

**Figure S2. Comparison of EFS and OS among patients with different factors**

(A-B) EFS and OS in stage III/IV and I/II patients; (C-D) EFS and OS in patients who received high-dose intensity and low-dose intensity of tislelizumab; (E-F) EFS and OS in patients who received high-dose intensity and low-dose intensity of chemotherapy.

Abbreviations: EFS, event-free survival; OS, overall survival; HR, hazard ratio; CI, confidence intervals.

**

**

**Figure S3. Comparison of OS among patients with different pathological stages**

(A) Comparison of OS among patients with different ypN stages; (B) Comparison of OS among patients with different ypT stages; (C) Comparison of OS among patients with ypN+, ypT+, and ypT0N0 stages.

**

**
